# Efficacy and Safety of Jueyin Granules for Patients with Mild-to-Moderate Psoriasis Vulgaris: Protocol for a Multicenter Randomized Placebo-Controlled Trial

**DOI:** 10.1155/2020/8942301

**Published:** 2020-04-09

**Authors:** Su Li, Cang Zhang, Hong-Ya Zhang, Meng Zhou, Si-Nong Wang, Rong Xu, Dong-Mei Zhou, Yun-Run Ji, Jing-Jing Lv, Qing-Feng Yin, Rui-Ping Wang, Wei Li, Yan-Ping Liu, Jian-Feng Wang, Bin Li, Xin Li

**Affiliations:** ^1^Department of Dermatology, Yueyang Hospital of Integrated Traditional Chinese and Western Medicine, Shanghai University of Traditional Chinese Medicine, Shanghai 200437, China; ^2^Department of Dermatology, Beijing Hospital of Traditional Chinese Medicine Affiliated to Capital Medical University, Beijing 100010, China; ^3^Department of Dermatology, The First Affiliated Hospital of Anhui University of Traditional Chinese Medicine, Hefei, Anhui 230031, China; ^4^Guangxi Zhuang Autonomous Region Institute of Dermatology Prevention and Control, Nanning, Guangxi 530007, China; ^5^Department of Dermatology, Affiliated Hospital of Gansu University of Traditional Chinese Medicine, Lanzhou, Gansu 730000, China; ^6^Jiangsu Famous Medical Technology Co. Ltd., Nanjing University of Traditional Chinese Medicine, Nanjing 210029, China; ^7^Office Clinical Research Center, Yueyang Hospital of Integrated Traditional Chinese and Western Medicine, Shanghai University of Traditional Chinese Medicine, Shanghai 200437, China; ^8^Institute of Dermatology, Shanghai Academy of Traditional Chinese Medicine, Shanghai 201203, China

## Abstract

*Introduction*. The etiology and pathogenesis of psoriasis are complex. Blood-heat syndrome is the core pathogenesis of psoriasis. Based on theories of Chinese medicine (CM), heat-clearing and blood-cooling (HCBC) are the primary treatment. Very few studies have investigated the pharmacological mechanism of the CM HCBC method for treating psoriasis. This multicenter randomized controlled trial will focus on treating psoriasis blood-heat syndrome with the HCBC method using Jueyin granules (JYKL). This will be an objective and standardized evaluation of the efficacy, safety, and reproducibility of the HCBC method to obtain objective evidence meeting international standards that aim to establish a clinical standard suitable for the popular application of CM for treating psoriasis. *Methods and Analysis*. A five-center randomized double-blind placebo-controlled clinical design will be used in this study. At least 196 participants will be randomly assigned to receive either JYKL or placebo treatment approximately 30 minutes after meals in the morning and evening (one sachet per time, twice daily for 8 consecutive weeks). The study duration will be 17 weeks, including 1 week of screening, 8 weeks of intervention, and 8 weeks of follow-up. The patients will be evaluated every 2 weeks, and the measures will be compared with baseline values. The primary outcome measure will be the psoriasis lesion area severity index. We will also observe the recurrence rate, body surface area, physician global assessment, dermatology life quality index, quality of life index, visual analogue scale score, CM symptom score, combined drug use, and adverse events. This trial is registered with NCT03961230.

## 1. Introduction

Psoriasis is a chronic inflammatory skin disease characterized by excessive proliferation of epidermal keratinocytes and hyperkeratosis caused by inflammatory infiltration in the dermis [1, 2]. Depending on race and region, 2–4% of the world's population suffers from psoriasis [3–5]. The prevalence of psoriasis is increasing without an increase in total cases. This may be due to increased awareness and earlier diagnosis [6]. There is currently slow recognition that psoriasis is a complex disease under a multifactor genetic model, which can be caused by the interaction of immunity, heredity, infection, and the environment [7, 8].

The etiology of psoriasis remains unclear. There are currently no fundamental drugs or treatment methods for psoriasis, which means that it is intractable and shows chronic recurrence, sometimes over an entire lifetime. Additionally, irregular or overtreatment by doctors causes tremendous financial burden and psychological stigma to patients and their families, seriously affecting quality of life [9, 10]. Therefore, a safe, effective, and economical treatment that results in a low recurrence rate is the most important goal for clinicians and researchers.

The treatment of psoriasis using modern medicine still focuses mainly on the immune system, systematic treatment, local symptomatic treatment, physical therapy, and similar methods. Most patients have achieved acceptable clinical results in the course of treatment, but side effects inevitably occur from medications, especially in systematic treatment. Therefore, a safe and effective Chinese medicine (CM) treatment has become the focus of addressing the difficulties treating psoriasis [11, 12].

CM has a long history in the treatment of psoriasis. CM advocates a holistic approach and attaches importance to the syndrome differentiation of viscera, qi, and blood. “External treatment must be based on the internal” is a fundamental principle of CM, including the treatment of psoriasis. CM medicinal compounds are multitargeted and multichanneled; they have been widely used clinically and are an essential part of CM. Furthermore, CM compounds have fewer side effects than modern medicine, are easier to manage, and are favored by patients because they can be highly tailored to each individual [13]. Blood-stage treatment is the primary syndrome-differentiation basis for psoriasis, and blood-heat syndrome is the most common syndrome type in the pathogenesis of psoriasis. Thus, heat-clearing and blood-cooling (HCBC) medicine has become the primary method to treat psoriasis using CM [14].

Representative prescription of Jueyin granules (JYKL) was created by Xiz Han, a well-known Chinese surgeon, in the 1950s and has been used by the Yueyang Hospital of Integrated Traditional Chinese and Western Medicine to clinically treat psoriasis for over 50 years [15]. The concoction is composed of seven herbs: abalone (*Haliotis diversicolor*), honeysuckle (*Lonicera japonica*), tree peony (*Paeonia suffruticosa*), Chinese foxglove (*Rehmannia glutinosa*), snake needle grass (*Hedyotis diffusa*), woad (*Isatis tinctoria*), and wild turmeric (*Curcuma aromatica*) (Table 1) [16].

Previous studies have shown that JYKL can reduce inflammation and keratinocyte proliferation and prevent the occurrence of psoriasis in animal models [17]. Moreover, JYKL's active ingredients, including shi cassia, *Lonicera japonica*, herba *Hedyotis diffusa*, Folium isatidis, and turmeric, have been demonstrated to have anti-inflammatory effects in both *in vitro* and *in vivo* models [18–23]. Peony bark has an inhibitory effect on the proliferation of HaCaT cells in models *in vitro* [24]. Although CM provides first-line drug treatment for millions of Chinese people, its application is often questioned in some Western medicine circles [25] possibly because most of the research on CM is limited to a single medical center, studies with small sample sizes, and a lack of rigorous randomized controlled trials (RCTs). Therefore, a rigorously designed RCT to investigate the efficacy and safety of JYKL is warranted.

The purpose of this research is to (1) enrich the scientific implication of CM in the treatment of psoriasis, (2) improve the CM methods for the clinical treatment of psoriasis, and (3) reduce the treatment cost of psoriasis.

## 2. Methods and Analysis

### 2.1. Study Design

This will be a multicenter randomized double-blind placebo-controlled clinical trial. It will aim to objectively standardize the evaluation of the clinical efficacy, safety, and recurrence of blood-heat syndrome of psoriasis vulgaris treated with HCBC in CM. The results will be helpful for establishing the clinical standard of traditional CM in the treatment of psoriasis. The trial has been registered at NCT03961230. The start and end of recruitment was planned for January 2019 and December 2021, respectively.

Prior to the commencement of the study, the investigators and researchers require uniform training to ensure that the medical staff involved in the study are fully aware of all aspects of the trial. This study will be performed in five centers in China: Yueyang Integrated Traditional Chinese and Western Medicine Hospital, Beijing Traditional Chinese Medicine Hospital, First Affiliated Hospital of Anhui University of Traditional Chinese Medicine, Affiliated Hospital of Gansu University of Traditional Chinese Medicine, and Guangxi Zhuang Autonomous Region Institute of Dermatology Prevention and Control. Competitive enrollment will be applied in all research centers to achieve the goal of 196 total participants. Each participant can be enrolled only once.

**Figure 1:**
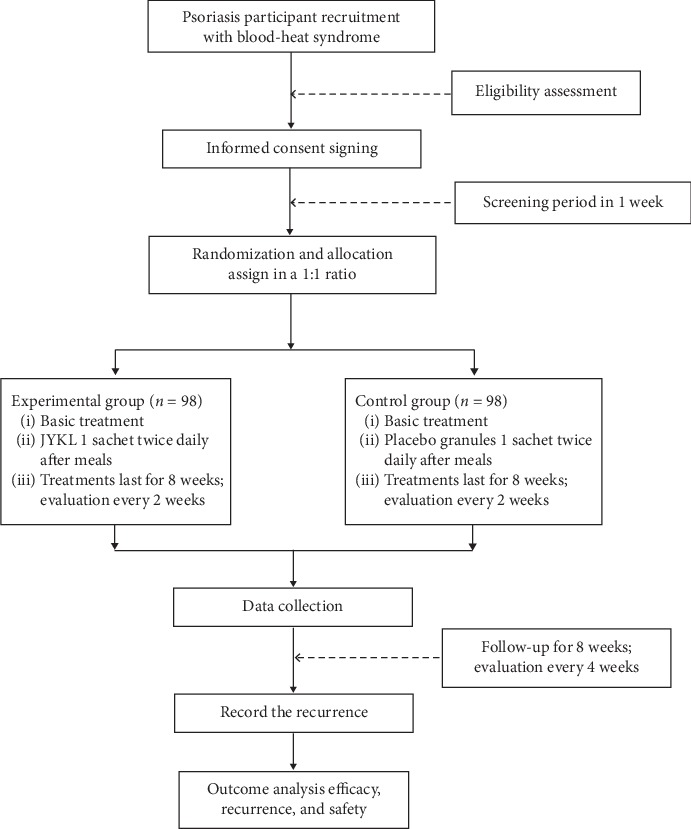
Flow diagram of the study process.

Five phases will be included in the study: screening/registration, allocation, treatment/intervention, end of the intervention, and follow-up. During the initial screening, participants will be recruited through dermatology clinics, where they will undergo a physical examination and eligibility assessment. According to the assessment results, a run-in period of up to one week will be required. All patients who are diagnosed with psoriasis with blood-heat syndrome will undergo laboratory blood testing, including a complete blood cell count, liver function test, renal function test, and a pregnancy test before study inclusion. Additionally, routine urine tests, vital sign monitoring, and physical examinations will be performed (Figure 1). Patients who are willing to join the clinical trial will receive study information and consent forms. The consent forms must be signed before a patient will be included in this study.

### 2.2. Eligibility Criteria

#### 2.2.1. Inclusion Criteria

Participants must meet all of the following criteria: (1) western medicine diagnostic criteria of drip-type or plaque psoriasis and CM syndrome diagnostic criteria of psoriasis blood-heat syndrome (this syndrome differentiation refers to the guiding principles for clinical research of new drugs of CM and the evidence-based clinical practice guide of CM for Psoriasis (2013 edition)) [26]; (2) skin lesions involving <10% body surface area (BSA) mainly located on the trunk, limbs, palms, soles, face, or scalp (vulvar area not included); (3) age 18–65 years; and (4) voluntary participation in this study and providing informed consent. There are no limitations based on sex or gender identity.

#### 2.2.2. Exclusion Criteria

Participants with be excluded if they meet any of these exclusion criteria: (1) presence of other active skin diseases that may affect the study; (2) history of systematic treatment with research drugs, biological agents, or immunosuppressants within the past 30 days; (3) history of receiving topical glucocorticoid, phototherapy, or similar treatment within the previous 14 days; (4) currently experiencing a severe and uncontrollable period of local or systemic acute or chronic infection; (5) presence of serious systemic diseases or the clinical detection indexes belonging to one of the following conditions—increase in glutamic pyruvic transaminase or glutamic oxaloacetic transaminase is more than 1.5 times of the upper limit of the normal value, an increase in creatinine, and any of the leading indicators of blood health (white blood cell count, red blood cell count, hemoglobin count, and platelet count) outside the normal range or other laboratory abnormalities as judged by researchers to be unsuitable for patients participating in this trial; (6) history of malignant tumors and patients with primary or secondary immunodeficiency and hypersensitivity; (7) major surgery within the previous 8 weeks or will be required during the study period; (8) currently pregnant or lactating; (9) history of alcoholism or drug abuse; (10) history (or family history) of severe mental illness; or (11) a family history of cancer.

### 2.3. Interventions

#### 2.3.1. Experimental Group: JYKL and Basic Treatment

The JYKL to be used in this study is produced and supplied by Hefei Sanjiu Pharmaceutical Co. Ltd.

Dosage and usage: JYKL will be administered approximately 30 minutes after meals in the morning and evening (one sachet per time, two times daily for consecutive 8 weeks). The specific administration method will be as follows: empty the content of one sachet into a mug, add 50 mL of warm water, stir until dissolved, and add another 50 mL of hot water. Consume all 100 mL of the liquid mixture as a single dosage. The participant will be asked to wear warm clothes.

#### 2.3.2. Control Group: Placebo and Basic Treatment

The placebo to be used in this study is also produced and supplied by Hefei Sanjiu Pharmaceutical Co. Ltd.

Dosage and usage: the placebo will be dosed and administered in the same manner as the JYKL.

Basic treatment is administered as follows:

1. Moisturizing: as a basic treatment, the use of a moisturizer is always necessary. Moisturizing and moisturizing agents will be applied to areas of the skin that do not erode late at night, including dry nonrash areas. A soft moisturizer without spices will be used; for sensitive and dry skin, a Yuze moisturizer is recommended.
2. Bathing and cleansing the skin: a reasonable bath which involves a quick overall rinse with warm water (35°C–39°C) for about 5 minutes once a day is indicated. The participant should then immediately apply moisturizing agents within 2 minutes after bathing to avoid skin dehydration. The use of alkaline detergents should be avoided.
3. Avoid aggravating factors: patients with food allergies should avoid eating allergy-aggravating foods to prevent inducing a psoriasis reaction.
4. Reasonable lifestyle: avoid staying up late, minimize stress, avoid spicy and irritating foods, stay physically active, and maintain regular bowel movements.
5. Adhere to reasonable treatment: doctors and patients should communicate regularly and establish mutual trust. Adhere to the treatment regimen for the best chance of alleviating the disease.

### 2.4. Combined Use of Drugs

1. Permissible combined treatment: participants may take drugs for other diseases (such as hypertension, diabetes, acute infection, or other reasons) if other drugs have not been confirmed as effective for treating psoriasis.
2. Prohibition of combined treatment: drugs that may induce or aggravate psoriasis may not be taken during the study.

### 2.5. Outcome Measures

#### 2.5.1. Primary Outcome

The primary outcome is the reduction in the Psoriasis Area Severity Index (PASI) score, which will be calculated as follows: PASI reduction = (PASI at baseline)–(PASI at week 8 of the treatment period).

The PASI score of the patients will be assessed every 2 weeks during the treatment period and every 4 weeks during the follow-up period. The main result of the RCT will be the percentage of patients whose PASI score decreased by more than 75% compared with the baseline at 8 weeks [2].

#### 2.5.2. Secondary Parameters

The secondary outcome measures include the following:

Body surface area (BSA): the percentage of BSA involved in psoriasis is estimated by the handprint method, in which the patient's entire palm (that is, the patient's fully outstretched palm, fingers, and thumb) represents about 1% of the total BSA [27].

Physician global assessment (PGA): the PGA is a 5-point scale that is used to reflect the overall assessment of erythema (E), infiltration (I), and desquamation (D) in psoriatic lesions [28].

Dermatology life quality index (DLQI): the DLQI is a 10-item questionnaire assessing the impact of skin disease on health-related quality of life (HRQOL). The total score ranges from 0 to 30; higher scores indicate a lower quality of life [29]. Foreign clinical studies have reported that its minimal clinically important difference (MCID) is 5 points [30]. That is, after treatment, DLQI is decreased by 5 points, which can be considered an improvement in quality of life and treatment efficacy.

Quality of life (QOL): QOL is a term used to describe a person's emotional, social, and physical health. In patients with psoriasis, QOL is similar to or worse than that of chronic diseases such as ischemic heart disease, hypertension, diabetes, and cancer [31]. The questionnaire consists of 15 questions, including five parts: daily activities, at work, personal relationships, leisure activities, and treatment. The answer to each question gives a score from 0 to 3. The choice of each question is “very many” (3 points), “a lot” (2 points), “a little” (1 point), and “no answer at all” (0 points) [32].

Visual Analogue Scale (VAS): VAS is an often-used tool to measure subjective phenomena, which has shown good reliability and validity in terms of assessment of pain. In the clinical study of psoriasis, it can be used as a tool to measure the degree of pruritus from 0 to 100 mm (with 0 being no pruritis and 100 being maximum pruritis) [33].

CM syndrome scoring scale (CMSSS): the CMSSS is used to assess changes in blood-hot syndrome-related symptoms during treatment [2].

The BSA, PGA, and VAS will be assessed every two weeks during the treatment period and every four weeks in the follow-up period. The DLQI, QOL, and CMSSS will be assessed by the patients every two weeks during the treatment period. In the follow-up period, the DLQI will only be assessed in the last week (week 16). Laboratory reports will also be monitored until the last visit (Table 2).

#### 2.5.3. Sample Size

The sample size for this study was calculated based on the expected value of the efficacy according to the clinical trial results and data analysis of recently published articles [34, 35]. The PASI-50 reached for experimental group and control group was 55.66% and 35.3%, respectively. The significance level (alpha) was set at 0.05, and the statistical power was 80%. Based on our calculation using the PASW statistical software (V.18.0), a sample size of 89 patients was required for experimental group and control group. Given a 10% loss to follow-up, we expect to require 98 participants in each group. As a result, this trial will require at least 196 participants in its current setup.

#### 2.5.4. Randomization, Allocation, and Blinding

This study will use central district stratification and block randomization. Random sequences will be generated by statistician researchers (implemented by the data Management Center of Jiangsu Famous Medical Technology Co. Ltd.). Eligible patients will be randomly assigned, in a 1 : 1 ratio, to one of the two groups (JYKL group or control group), aiming to balance baseline characteristics between the groups.

Throughout the study, participants, subjects, and study monitors will be blinded. Research drug random coding will be the only information linked to the group allocations. The random code will be maintained by Jiangsu Famous Medical Technology Data Management Center to ensure concealment. For the purpose of the statistical analysis, the JYKL intervention will be considered the exposure. If a serious adverse event occurs and the patient needs to be rescued, making it is necessary to know the drug used by the patient, the person in charge of each center shall decide to open the blindness and open the corresponding emergency letter. As soon as the corresponding numbered emergency letter is opened, the case will not be included in the study.

#### 2.5.5. Safety Evaluation

Vital signs, adverse events, and severe adverse events will be evaluated before treatment at the following points: week 0, week 2, week 4, week 6, week 8, and during week 16 follow-up period. We will evaluate the blood routine, blood biochemistry, routine urine, and physical examination during the screening period, the 8^th^ week of treatment, and the follow-up period (16^th^ week). We will screen for pregnancy during the initial participant screening (Table 2).

#### 2.5.6. Recurrence Evaluation

All patients who complete the treatment will be followed up for 8 weeks, and the psoriasis area and a PASI score will be recorded. A PASI score that exceeds the baseline score at the time of admission, new pustules, or erythroderma will be considered recurrence. During the follow-up period, if a patient is assessed as having recurrence, the patient will be able to decide whether to end the follow-up and start further treatment.

#### 2.5.7. Time Window

The indices of blood routine, blood biochemistry, and routine urine will be considered valid when measured in the first 7 days before the baseline period; 14 ± 3 d is acceptable for the measurements to be taken every 2 weeks during the treatment period, while 28 ± 5 d is acceptable for the measurements to be taken every 4 weeks during the follow-up period.

#### 2.5.8. Drug Quality Control

The JKYL medicinal (treatment and placebo) ingredients will be received from the same place of origin and the same season of the year. They will be purchased in full quantity and reserved after a quality inspection. The preparation methods for both groups' medicines shall be the same.

#### 2.5.9. Timeline

Recruitment began in September 2019 and is expected to end by December 2021. Table 2 provides a research timetable for registration, intervention, and evaluation.

#### 2.5.10. Data Collection and Management

After the test scheme is determined, a professional statistician shall be responsible for formulating the statistical analysis plan in consultation with the principal researchers using SAS statistical software and the data network platform designed by Jiangsu Famous Medical Technology Co. Ltd. Data Management Center.

#### 2.5.11. Statistical Analysis

The data analysis will be performed using SAS software (version 9.2). We will describe the data by using frequency counts and proportions (prevalence) for qualitative variables and means. Standard deviation (SD) will be used for normally distributed quantitative variables, while median and interquartile range (Q1 and Q3) will be used for nonnormally distributed quantitative variables. We will apply the chi-square test to examine the differences between the experimental and control groups for qualitative variables, the *t*-test to examine the differences between two groups for normally distributed quantitative variables (the homogeneity of variance will be tested between groups, and the Satterthwaite approximation will be used to correct the variance when the variance is uneven), and the Wilcoxon rank-sum test for nonnormally distributed quantitative variables. A generalized linear model will be applied for quantitative variables with repeated measures. In this study, *p* values < 0.05 (two-tailed) will be considered statistically significant.

## 3. Discussion

Psoriasis is a chronic immunity-related inflammatory skin disease [36, 37] that affects about 100 million people worldwide [38]. Due to its high prevalence, the World Health Organization considers psoriasis a serious global problem [38]. In recent years, psoriasis has become increasingly considered a disease that not only affects the skin but also multiple systems [39]. Due to the influence of polygenic linkage, patients with psoriasis are prone to metabolic syndrome, cardiovascular disease, diabetes, and other systemic diseases. Furthermore, the severity of psoriasis is significantly correlated with comorbidities [40, 41]. Although psoriasis is not an immediately life-threatening disease, it still reduces the quality of life of patients and brings a heavy economic burden on society [42]. Additionally, some patients with psoriasis, especially women, will have quite serious psychological disorders that will undoubtedly aggravate psoriasis, thus further reducing quality of life [43, 44]. The cause of this disease is currently unclear, and there is no treatment with a definite curative effect or a better prognosis. On the one hand, it causes patients to blindly pursue quick radical cures, believe false propaganda, and excessively trust unproven drugs and solutions, resulting in unreasonable, irregular treatment that extends and aggravates the disease. On the other hand, it encourages clinicians to give full play to their strengths, explore a wide range of ideas, actively seek new treatment methods, research and develop new drugs, and explore new treatment methods. We have made progress in understanding the pathogenesis of psoriasis, but the efficacy of modern therapies is exceedingly limited. People are seeking new drugs to treat psoriasis, but because of the prohibitive cost, many patients cannot afford new treatments [45]. Therefore, effectively treating psoriasis and improving patient quality of life have become the focus of dermatological research.

After the Ming and Qing dynasties, “blood-stage treatment” became the primary theoretical basis of syndrome differentiation and the treatment of psoriasis. The epidemiological study of many large samples revealed that the incidence of “blood-heat syndrome” ranked first among all types of psoriasis. JYKL is a CM compound developed based on Professor Xia Han's experimental prescription “XueRe No. 1” and many years of clinical experience. JYKL is clinically used to treat psoriasis vulgaris, especially the blood-heat type. Although traditional medicine provides front-line pharmacotherapy for millions of Chinese individuals, its application is often viewed with skepticism by the Western medical establishment. Therefore, it is necessary to scientifically verify that treatment using JYKL has a curative effect on the psoriasis with blood-heat syndrome and verify that blood-heat syndrome is the core problem to be solved.

Based on the clinical experience with “blood-stage treatment,” this project will objectively and normatively evaluate the clinical efficacy, safety, and control of relapse of the blood-heat syndrome in psoriasis vulgaris treated with JYKL (the representative CM prescription for blood-heat syndrome) through a multicenter double-blind RCT. This will further validate the CM treatment scheme for blood-heat syndrome and psoriasis vulgaris, thereby providing high-level evidence through rigorous research.

## Figures and Tables

**Table 1:** Ingredients of Jueyin granules (intervention drug) with English translations.

Common English name	Chinese pinyin	*Genus species* (Latin name)	Plant part
Abalone	*Shi Jue Ming*	*Haliotis diversicolor*	Shell
Honeysuckle	*Jin Yin Hua*	*Lonicera japonica*	Buds or first-blooming flowers
Tree peony	*Mu Dan Pi*	*Paeonia suffruticosa*	Root cortices
Chinese foxglove	*Sheng Di Huang*	*Rehmannia glutinosa*	Root
Snake needle grass	*Bai Huang Sheng Cao*	*Hedyotis diffusa*	Whole grass
Woad	*Da Qing Ye*	*Isatis tinctoria*	Leaf
Wild turmeric	*Yu Jin*	*Curcuma aromatica*	Rhizome

**Table 2:** Schedule for enrollment, intervention, and assessment.

Activity	Phase	Screen/enroll	Allocation	Treatment/intervention				Intervention end	Follow-up	
	Time points	Week 0		Weeks 1-2	Weeks 3-4	Weeks 5-6	Weeks 7-8		Week 12	Week 16
Screening/enrollment	Eligibility screening	●	●							
	Acquisition of informed consent	●								
	Clinicopathological evaluation	●								
	Medical history recorded	●								
	Enrollment	●								
	Random allocation		●							
	Biological specimen collection		●	●	●	●	●	●	●	●

Treatment/intervention	Basic treatment + JYKL		★-------------------------------------------------------------------------★								
	Basic treatment + placebo granules		☆-------------------------------------------------------------------------☆								

Outcome assessment	PASI score	●	●	●	●	●	●	●	●	●
	Affected BSA	●	●	●	●	●	●	●	●	●
	PGA score	●	●	●	●	●	●	●	●	●
	DLQI score		●	●	●	●	●	●		●
	QOL score		●	●	●	●	●	●		●
	VAS score		●	●	●	●	●	●	●	●
	CM syndrome		●	●	●	●	●	●		●
	Recurrence								●	●

Safety assessment	Vital signs	●	●	●	●	●	●	●		●
	BR	●					●	●		●
	BB	●					●	●		●
	RUT	●					●	●		●
	DC	●	●	●	●	●	●	●		●
	PT	●								
	PE	●					●	●		●
	AEs		●	●	●	●	●	●		●
	Severe AEs		●	●	●	●	●	●		●

✩, intervention in the control group; ★, intervention in the experimental group; ●, what needs to be done in the corresponding time period. JYKL, Jueyin granules; PASI, Psoriasis Area and Severity Index; BSA, body surface area; PGA, physician global assessment; DLQI, dermatology life quality index; QOL, quality of life; VAS, Visual Analogue Scale; CM, Chinese medicine; BR, blood routine; BB, blood biochemistry; RUT, routine urine test; DC, drug combination; PT, pregnancy test; PE, physical examination; AE, adverse event.

## Data Availability

The clinical trial data used to support the findings of this study have not been made available because the status of this trial is recruiting.
